# Potent Restriction of Sexual Zika Virus Infection by the Lipid Fraction of Extracellular Vesicles in Semen

**DOI:** 10.3389/fmicb.2020.574054

**Published:** 2020-09-29

**Authors:** Ruofan Wang, Germán G. Gornalusse, Yeseul Kim, Urvashi Pandey, Florian Hladik, Lucia Vojtech

**Affiliations:** ^1^ Department of Obstetrics and Gynecology, University of Washington, Seattle, WA, United States; ^2^ Division of Allergy and Infectious Diseases, Department of Medicine, University of Washington, Seattle, WA, United States; ^3^ Vaccine and Infectious Disease Division, Fred Hutchinson Cancer Research Center, Seattle, WA, United States

**Keywords:** Zika virus, flavivirus, semen, extracellular vesicles, exosomes, female, genital, vagina

## Abstract

Sexual Zika virus (ZIKV) transmission from men to women occurs less frequently than the often-detected high viral loads in semen would suggest, but worries that this transmission route predisposes to fetal damage in pregnant women remain. To better understand sexual ZIKV pathogenesis, we studied the permissiveness of the human female genital tract to infection and the effect of semen on this process. ZIKV replicates in vaginal tissues and primary epithelial cells from the vagina, ectocervix, and endocervix and induces an innate immune response, but also continues to replicate without cytopathic effect. Infection of genital cells and tissues is strongly inhibited by extracellular vesicles (EV) in semen at physiological vesicle-to-virus ratios. Liposomes with the same composition as semen EVs also impair infection, indicating that the EV’s lipid fraction, rather than their protein or RNA cargo, is responsible for this anti-viral effect. Thus, EVs in semen potently restrict ZIKV transmission, but the virus propagates well once infection in the recipient mucosa has been established.

## Introduction

Since the emergence of Zika virus (ZIKV) in the Americas, millions of people have been infected and transmission of virus continues in many areas (Siedner et al., 2018). Though the majority of cases are asymptomatic or cause only mild symptoms, serious conditions including myelitis, meningoencephalitis, and Guillain-Barré syndrome afflict some infected adults (Cao-Lormeau et al., 2016; Carteaux et al., 2016; Mécharles et al., 2016). Most concerning is the ability of ZIKV to infect developing fetuses in utero and cause fetal demise or congenital Zika syndrome, a spectrum of anomalies, and disabilities that results from damage to the nervous system in 5–42% of pregnancies with ZIKV infection, depending on how outcomes are defined (Brasil et al., 2016; Chimelli et al., 2017; del Campo et al., 2017; Moore et al., 2017; Musso et al., 2019).

Though typically transmitted *via* the bite of an infected mosquito, sexual transmission of ZIKV among women and men who have sex with men, including from asymptomatic men, has been well-documented. Semen from infected men can harbor high concentrations of ZIKV viral RNA, up to 10^9^ copies per ml, which can persist up to 9 months after the onset of symptoms (de Laval et al., 2017; Mead et al., 2018; Medina et al., 2019). Early in the epidemic, this finding led to great concern over sexual transmission of ZIKV (Mansuy et al., 2016). Consistent with a higher risk of infection in women due to sexual transmission from men, incidence rates of ZIKV were 90% greater in women than men in one study in Rio de Janeiro (Coelho et al., 2016). More recent epidemiological and mathematical modeling data suggest that though sexual transmission alone likely cannot sustain the epidemic (Yakob et al., 2016), sexual transmission of ZIKV may account for between 3 and 15% of infections (with upper confidence ranges of 30–46%; Gao et al., 2016; Towers et al., 2016; Sasmal et al., 2018). Yet, only a small proportion of semen samples with detectable viral loads actually contains culturable virus: 8 of 78 tested samples in one study, and 3 of 78 in another, and all from samples with >10^6^ genome copies per milliliter, with culturable virus more frequent in men with the highest viral loads (Mead et al., 2018; Medina et al., 2019). Thus, the risk of sexual transmission from men with moderate or low viral loads appears to be small. Nevertheless, the consequences to fetuses following sexual ZIKV transmission may be enhanced compared to mosquito-borne transmission. In a macaque model comparing vaginal and subcutaneous exposure, vaginal challenge resulted in preferential ZIKV replication in the reproductive tract and induced enhanced local and systemic inflammation (Carroll et al., 2017). In murine models, the risk to fetuses of greater infection rates, higher viral loads, and morbidity and mortality, is increased following vaginal, compared to subcutaneous or intraperitoneal exposure to ZIKV (Yockey et al., 2016; Winkler et al., 2017; Duggal et al., 2018). These findings suggest that the pathogenesis of ZIKV varies by the route of transmission and highlight the need to thoroughly understand sexually transmitted ZIKV infections. Notably, semen, which is co-delivered to the vaginal tract at the same time as virus, was absent from these studies, and it is important to understand how factors in semen influence ZIKV infection.

Our study was undertaken to investigate the susceptibility of the female genital mucosa to ZIKV infection, and how the immune system responds. Utilizing our well-characterized model of *ex-vivo* vaginal tissue (Hladik et al., 2007; McElrath et al., 2010; Ballweber et al., 2011), as well as primary untransformed human genital tract cell lines (Hladik et al., 2015), we found that ZIKV replicates efficiently in vaginal, ectocervical, and endocervical epithelial cells, and vaginal tissue explants, without causing cell death. These cells mount an innate immune response characterized by the induction of high levels of interferon β, interferon λ, and chemokines. In addition to describing the early pathogenesis of ZIKV in the female genital mucosa, we investigate how extracellular vesicles in semen (SEV), co-delivered with virions during sexual transmission, affect ZIKV pathogenesis. SEV are small lipid membrane-bound particles containing bioactive nucleic acids and proteins, which are released by cells of the male genital tract into seminal fluid. We report that SEV inhibit ZIKV infection, as published by one other group (Müller et al., 2018). We extend these findings to show that SEV inhibit infection *via* their lipid content, and that naked liposomes also potently inhibit ZIKV infection. Furthermore, we report that SEV and liposomes do not block cellular phosphatidylserine receptors but rather cause degradation of the ZIKV genome. Suppression of viral infection by components of semen likely explains why sexual transmission of ZIKV is relatively rare despite readily detectable viral loads found in semen and the ability of genital epithelial cells to sustain infection.

## Materials and Methods

### Isolation and Culture of Primary Epithelial Cells

Discarded vaginal and cervical tissues were collected from healthy donors during repair surgeries and immediately transported to the laboratory on ice. Tissue samples were collected under IRB-approved protocols at the University of Washington and Fred Hutchinson Cancer Research Center.

Cultures of primary untransformed epithelial cells were generated as we previously reported (Hladik et al., 2015), using a protocol originally established by Chapman et al. (2010). Briefly, to isolate epithelial layers, tissue chunks were trimmed of excess stroma and were treated with 20 ml Dispase II (12.5 U/ml) in Hank’s balanced salt solution overnight in 4°C fridge. Epithelial sheets were separated from underlying stroma with forceps under stereoscope in the morning. Epithelial sheets were transferred to a six-well plate and cultured in F-media (3:1 v/v of F12 media and DMEM high glucose containing 5% heat-inactivated fetal calf serum, 0.4 μg/ml hydrocortisone, 5 μg/ml insulin, 8.4 ng/ml cholera toxin, 10 ng/ml epidermal growth factor, 24 μg/ml adenine, 100 U/ml penicillin/streptomycin, and 2 mM L-glutamine) at 37°C incubator with 5% CO_2_ for more than 48 h. Excess epithelial sheets were discarded once cells were attached to the plate. Attached cells were washed with DPBS and dissociated with 10 ml of 0.25% Trypsin-EDTA. The dispersed cells were poured through a 100-μm cell strainer into a 50-ml tube and pelleted by centrifugation. Cells were further sub-cultured with irradiated 3T3 fibroblast feeder cells and Rho-kinase inhibitor Y-27632 (3.33 ug/ml; Tocris Bioscience) to inhibit dedifferentiation in F medium for at least four passages before being considered established.

### Zika Stocks

Zika virus strain VR-1848 was purchased from the American Type Culture Collection (ATCC). Viral stocks were generated by infecting Vero cells (obtained from the laboratory of Dr. David Koelle, University of Washington) at 70% confluence with an approximate multiplicity of infection (MOI) of 0.1 of virus in Dulbecco’s Modified Eagle Media for 1.5 h, then removing inoculum and replacing with VP-SFM serum-free low protein medium (Gibco). Supernatants were harvested when Vero cells had visible cytopathic effects, at 4- or 5-days post infection. Stocks were centrifuged at 350 × *g* for 10 min to pellet cell debris, then concentrated 7-fold in Centricon plus 70–100 kDa cutoff centrifugal filter unit. Mock infected Vero supernatants were generated and concentrated in the same way. Stocks were aliquoted and stored at −80°. All experiments were done with virus passaged twice in Vero cells. Viral titer was determined as plaque-forming units (PFU) per ml as described below.

### Plaque Assays

Five hundred thousand Vero cells were plated per well on six-well plates in D10 media (DMEM, 10% FBS, 2 mM L-glutamine, 50 U/ml penicillin, and streptomycin) 1 day prior to infection. Virus-containing culture media were diluted in DMEM media then added to the Vero cells (1 ml/well). Cells were incubated for 1.5 h then inoculum was removed. D10 media with 0.4% agarose was added to each well (2.5 ml/well). After agarose solidified for 5 min, plates were incubated at 37°C for 3–5 days until the plaques became visible. Plates were fixed with 10% neutral buffered formalin for at least 2 h in room temperature. Agarose was removed after fixation and 0.5% crystal violet in 10% ethanol was added to the wells for staining then washed off with water prior to counting plaques by eye.

### Epithelial Cell Infections

Twenty-four hours prior to infection, irradiated 3T3 fibroblasts were removed from epithelial culture flask by adding 10 ml of versene [0.48 mM EDTA in phosphate-buffered saline (PBS)] followed by 10 min of incubation at 37°C. Epithelial cells were then dissociated with 10 ml of 0.25% trypsin/EDTA, counted, and plated in 12-well plates with 100,000 cells/well in F-media and 10 μM ROCK inhibitor. On the day of infection, Zika virus (VR-1848; Vero mock control), diluted in plain DMEM to 350 μl with an MOI of 1, was added to the cells and incubated for 1.5 h. Inoculumwas aspirated and washed away after incubation and replaced by F media with ROCK inhibitor. Culture media was harvested at 3, 24, 48, 72, and 144 hpi and PFU/ml was determined by plaque assay, as explained above. Cells were lysed in 350 μl of RLT lysis buffer (Qiagen) containing 1% beta-mercaptoethanol for RNA analysis or fixed with 4% paraformaldehyde for 15 min for immunofluorescence staining.

### Viral RNA Quantification With Digital Droplet PCR

To quantify ZIKV copy number, we used a two-step reverse transcription droplet digital PCR (RT-ddPCR) approach to detect ZIKV genomes (positive strand). RNA of infected cells was extracted with Qiagen RNeasy Plus Mini kit (Qiagen) according to manufacturer’s instructions and eluted with 30–40 μl RNase-free water. Genomic DNA was eliminated using the gDNA eliminator columns that are included in the kit. RNA concentration was quantified with NanoDrop spectrometer (Thermofisher). Two hundred nanograms of extracted RNA were used as template for cDNA synthesis with High-Capacity cDNA Reverse Transcription Kit (Applied Biosystem) and random hexamers. Upon completion of cDNA synthesis, samples were diluted 1:10 by adding 180 μl of molecular biology grade water. Five microliter of 1:10 diluted cDNA was used for ddPCR droplet generation. ddPCR reaction was done in a total volume of 22 μl of a mixture that contained 11 μl of ddPCR Supermix for Probes with no dUTP (BioRad), 1.1 μl of 20 × 6-carboxyfluorescein (FAM)-labeled target ZIKV quantitative PCR (qPCR) probe/primers, 1.1 μl of 20X HEX-labeled housekeeping RPP30 gene-specific Taqman gene expression probe/primers (Integrated DNA Technologies) and 5 μl of diluted cDNA. The primers and probe to detect ZIKV RNA were as follows: (Forward primer: 5'-CCG CTG CCC AAC ACA AG-3', reverse primer: 5'CCA CTA ACG TTC TTT TGC AGA CAT-3'), (Probe: 5'−/56-FAM/AGC CTA CCT/ZEM/TGA CAA GCA GTC AGA CAC TCA A/−3'), and housekeeper gene RPP30 (IDT DNA technologies assay ID Hs.PT.58.19785851). Each assembled ddPCR reaction mixture was loaded in duplicate into the sample wells of an eight-channel disposable droplet generator cartridge (BioRad) and droplet generation oil (BioRad) was added. After droplet generation, the samples were amplified to the endpoint in 96-well PCR plates on a conventional thermal cycler (C1000, Biorad) using the following conditions: denaturation/enzyme activation for 10 min at 95°C, 40–60 cycles of 30 s denaturation at 94°C and 60 s annealing/amplification at 60°C, followed by a final 10 min incubation step at 98°C. After PCR, the droplets were read on the QX100 Droplet Reader (BioRad). Analysis of the ddPCR data was performed with QuantaSoft analysis software version 1.3.1.0 (BioRad). A non-template control well containing ddPCR reaction mix but no cDNA was included to adjust the reaction threshold. Droplets positive for viral RNA were normalized to housekeeper gene copy number.

### Immunofluorescence

Duplicate well of cells was infected with ZIKV as above and processed for imaging at 72 and 144 hpi. Cells were washed, fixed with 10% neutral buffered formalin, permeabilized with 0.3% Triton X-100 in PBS for 20 min, and blocked with 5% BSA in PBS overnight at 4°. Then cells were stained with anti-flavivirus E protein antibody 4G2 conjugated to AF647 (Novus biologicals catalog NBP2-52709AF647) at 1:500 for a minimum of 1 h. After washing, cells were counterstained with DAPI and imaged using an EVOS FL cell imaging system (ThermoFisher). DAPI stained nuclei were counted using the automatic cell counting feature in the EVOs software (software revision 32,044), and 4G2 positive cells in the same fields were counted by hand. At least 10 fields and 1,000 DAPI positive cells were counted for each well. For receptor transfection experiments, DC-SIGN transfected cells were stained with a mouse antibody against DC-SIGN conjugated to AF647 (Biolegend 33011). TIM-1 transfected cells were stained with goat-anti-TIM-1 antibody (R&D systems AF1750), then with an anti-goat secondary antibody conjugated to AF488 (Invitrogen A-11055). Cells were counterstained with DAPI and imaged on an EVOS FL system as above.

### Tissue Infections

Vaginal tissues from surgeries were processed as described above for epithelial cell line generation. One or two sheets were added per well in a 48-well dish (depending on amount of tissue available) and infected with 500,000 PFU of ZIKV in DMEM at 37° overnight. The next morning, inoculum was removed and centrifuged at 300 × *g* to recover isolated cells. Sheets and isolated cells were washed 3x with PBS, retaining cells each time. For wells analyzed at day 1, sheets and cells were pooled and stored in 700 μl RNAlater (Ambion) at −20° prior to RNA extraction. For 5-day cultures, cells were added back to individual wells with RPMI media supplemented with 10% human AB serum, 100 U/ml penicillin/streptomycin, and 2 mM L-glutamine. At 5 days post infection, cells and sheets were washed 3x with PBS then stored and analyzed as for day 1-well.

### RNA Extraction From Tissues

Epithelial sheets and cells in RNAlater were pelleted by centrifugation at 3,000 × *g* for 5 min, then 600 μl of buffer RLT (Qiagen) was added. Tissues were homogenized on ice with a probe homogenizer for 5–10 s then pulled through a 20-gauge syringe seven times to shear genomic DNA. Lysates were incubated at room temperature for 10 min then 980 μl H_2_O and 20 μl proteinase K (Qiagen) added. Lysates were incubated for 15 min at 55° with shaking, then centrifuged at 10,000 × *g* for 3 min. Five-tenths volume of ethanol was added to the supernatants, and RNA extracted using Qiagen RNeasy kits with on-column DNAse digestion according to manufacturer’s instructions.

### Immune Gene Profiling

cDNA was generated as described above and it was diluted 1:10 with water. Five mictoliter was used in 15 μl final qPCR reaction using PrimeTime Gene Expression Master Mix (Integrated DNA Technologies) and primer probe sets specific for each gene in custom 96-well TaqMan array plates (Table 1). Thermocycling was done on a QuantStudio 5 (Applied Biosystems). The relative expression of each gene was calculated using the ΔCt method, and the geometric mean of the reference genes included in the array (i.e., 18srRNA, HPRT, GAPDH, and GUSB). Fold change compared to mock infected epithelial cells was calculated using the ΔΔCt method.

**Table 1:** Immune gene assays.

Gene	Assay ID (ThermoFisher)
18srRNA	Hs99999901_s1
GAPDH	Hs99999905_m1
HPRT	Hs99999909_m1
GUSB	Hs99999908_m
CXCL9	Hs00171065_m1
CXCL10	Hs00171042_m1
CXCL11	Hs00171138_m1
IFNA1	Hs03044218_g1
IFNB1	Hs01077958_s1
IFNL2	Hs00820125_g1
CCL2	Hs00234140_m1
IL1B	Hs01555410_m1
IL1RN	Hs00893626_m1
IL10	Hs00961622_m1
CCL5	Hs00982282_m1
IRF7	Hs01014809_g1

### Meso Scale Discovery Immunoassays for Secreted Cytokines

Supernatants from infected cell cultures were collected and frozen at −20°. Assay plates and reagents were purchased from Meso Scale Discovery. Plates were prepared per manufacturer’s instructions. Twenty-five microliters of standards or undiluted cell culture supernatant were added to wells for 1 h at room temperature prior to washing and addition of detection antibodies. Plates were read on MSD plate reader (MESO QuickPlex SQ 120). Samples were run in duplicates and protein concentrations were determined using MSD Discovery Workbench 4.0 analysis software. The light intensities from samples were interpolated using a four-parameter logistic fit to a standard curve of electrochemiluminescence generated from known concentrations. Results were exported and analyzed using R studio 1.1.463.

### Cell Viability Assay

Epithelial cells were plated at 10,000 cells per well in 96-well opaque white plates. The next day, at least six wells per cell type were infected (or mock infected), with ZIKV at an MOI of 1 for 1.5 h then cultured in complete F media as described above for 72 h. Then 100 μl of supernatant was removed, and 100 μl of reconstituted Viral ToxGlo reagent was added to each well, per manufacturer’s instructions (Promega). Luminescence was read on a Molecular Devices iD5 plate reader and results normalized to the average of mock infected control cells.

### Isolation and Quantification of Seminal Extracellular Vesicles

Small SEV, defined as below 220 nm in size, were purified from semen as previously described (Vojtech et al., 2014). Briefly, following liquefaction, seminal plasma was separated from the cell fraction by centrifugation and cell debris removed by 0.45 and 0.22 μm syringe filtration (Millex HA). SEV were purified by ultracentrifugation at 100,000 × *g* in a swinging bucket rotor over 20 mM Tris/30% sucrose/deuterium oxide cushion (pH 7.4) for 2 h then over a 20 mM Tris/25% sucrose/deuterium oxide cushion (pH 7.4) for 14 h (Lamparski et al., 2002). Supernatants above the sucrose cushions, containing vesicle-depleted seminal plasma were pooled from both ultracentrifuge spins. The sucrose cushions containing SEV were pooled and washed with 30 ml of Dulbecco’s PBS by centrifugation at 2400 × *g* in an Amicon Ultracel 100 kDa cellulose centrifugal filter and concentrated to a final volume of 425 μl–3.2 ml per donor. SEV were stored at −80°C. The SEV pool consisted of vesicles purified from five different semen donors. In previous publications (Vojtech et al., 2014), we have more extensively characterized SEV preparations made with the same protocol, including western blotting for common exosome markers and characterization of RNA content of these EV, in accordance with MISEV recommendations (Théry et al., 2018). Transmission electron micrographs of isolated SEV are in Supplementary Figure 1.

Concentration and size distribution of SEV from individual donors and from SEV pools were measured by nanoparticle tracking analysis (NTA) using a Nanosight NS300 instrument (Malvern) according to the manufacturer’s instructions. SEV-depleted semen was also analyzed using NTA. In brief, SEV or semen plasma samples were vortexed and serially diluted to a final dilution of 1:6,000–1:15,000 in filtered molecular grade PBS. Blank filtered PBS was run as a negative control. Each sample analysis was conducted for 60 s using Nanosight automatic analysis settings. Samples were evaluated in triplicate and concentration values were averaged. We found that 90% of EV was recovered in the semen EV preparations, while 10% of detectable particles remained in seminal supernatants.

### Liposome Generation

Lipids and cholesterol were purchased from Avantii Polar Lipids and dissolved in chloroform, then mixed in a rotovap cylinder according to the ratios shown in Table 2. Chloroform was evaporated under vacuum using a rotovap at 50° for 30 min. Evaporated solids were resuspended in 2.5 ml of room temperature PBS (approximate concentration of lipids: 5 mg/ml) with pipetting and subjected to sonification for 90 s in a water bath sonicator. Liposomes were freeze-thawed in a dry-ice ethanol bath twice, and then filtered through 0.45 μm and 0.22 μm syringe filters before aliquoting and freezing at −80C. One aliquot was thawed and checked for size distribution and concentration using the Nanosight as described for SEV. Aliquots of liposomes were thawed and used within 1 day and never re-frozen.

**Table 2:** Liposome preparation ratios.

Name	Catalog number	g/mol	Use
Cholesterol	70,000	386.965	6.45 mg = 16.67 μmol
Sphingomyelin	860061C	710.965	0.284 ml = 4 μmol
1-palmitoyl-2-oleoyl-sn-glycero-3-phosphoethanolamine	850757C	717.531	0.108 ml = 1.5 μmol
1-stearoyl-2-oleoyl-sn-glycero-3-phospho-L-serine (sodium salt)	840039C	811.534	0.101 ml = 1.25 μmol
L-α-phosphatidylcholine	840051C	770.123	0.077 ml = 1 μmol
L-α-phosphatidylinositol-4-phosphate (ammonium salt)	840,045	974.764	0.244 ml = 0.25 μmol

### Infection Experiments With Whole Seminal Plasma, SEV, Liposomes, and Recombinant Gas6

Cells were spun out of whole semen by centrifugation at 1,000 × *g* for 20 min. Seminal plasma was combined from five semen donors. Based on average concentration of SEV per ml of input seminal plasma, we estimate that at 5% volume of seminal plasma in viral incubation solutions contains approximately 10^6^ SEV per PFU of ZIKV. Therefore, we used 5% volume of whole semen or vesicle depleted seminal plasma in infection experiments. Vesicle-depleted seminal plasma was the supernatant left after SEV purification, as outlined above. Whole seminal plasma, vesicle-depleted semen, isolated SEV, or liposomes were mixed at ratios of 10^6^, 10^5^, or 10^4^ vesicles per PFU of ZIKV in a minimal reaction volume, which was approximately 40 μl per condition. These mixtures were incubated at 37° for 1 h, and then added to plain DMEM to make viral inoculum. Cell lines or tissues were infected as described above. For experiments where cells were pre-incubated with SEV, the SEV were mixed with plain DMEM to 350 μl and placed on cells for 1 h at 37°, and then ZIKV was added to the wells for an additional 1.5 h at 37° before removal of entire inoculum. Cells or tissues were washed with PBS before adding RTL buffer lysis containing 1% V/V of β-mercaptoethanol. Lysates were kept at −80°C until time of analysis.

For heat-treatment experiments, SEV were treated by heating at 95°C for 8 min then placed on ice. The same volumes of untreated and heat-treated SEV were mixed with ZIKV virions (for a 10^6^ ratio of SEV:virion) and used to infect cells as previously described.

For experiments where Gas 6 was added to the cultures, recombinant Gas6 (R&D 885-GSB-050 lot DFGX0519091) was added at indicated concentrations to the cells at the time of addition of ZIKV alone or ZIKV that had been pre-incubated with SEV or liposomes for 1 h at 37°. Solutions were left for 1.5 h at 37° before removal of entire inoculum. Cells or tissues were washed with PBS before adding RTL buffer lysis containing 1% V/V of β-mercaptoethanol. Lysates were kept at −80°C until time of analysis.

### HEK293 Transfection Experiments

HEK293 cells were plated at 95,000 cells per well in 48-well poly-d-lysine coated plates 1 day prior to transfection. Cells were transfected with lipofectamine (Life Technologies) according to manufacturer’s instruction using 500 ng of DNA in blank DMEM per well. TIM-1 expression plasmid was purchased from Sino Biological (catalog HG11051-UT, TIM-1 cDNA ORF clone human untagged). The DC-SIGN plasmid was obtained from the NIH AIDS reagent program, NIAID, NIH: Human DC-SIGN Expression Vector (pcDNA3-DC-SIGN) from Drs. S. Pöhlmann, F. Baribaud, F. Kirchhoff, and R.W. Doms (cat# 5,444; Pöhlmann et al., 2001). Efficiency of transfections was checked by immunofluorescence and flow cytometry; DC-SIGN was expressed by 32% of cells and TIM-1 by 72% of cells (representative expression from three separate experiments). Twenty-four hours post transfection, cells were exposed to ZIKV alone or pre-incubated with SEV or liposomes at an MOI of 1 for 1.5 h in blank DMEM. Inoculum was removed an complete HEK293 media added, cells were left for an additional 24 h prior to lysing for RNA extraction and quantification of ZIKV genomes by RT-ddPCR.

### RNAse Protection Assay

Ten microliters of ZIKV stock containing 73,000 PFU were incubated with 7.3 × 10^10^ SEV, liposomes, or PBS for 20 μl final volumes, for 1 h at 37°. To half the samples, one microliter of an RNAse A/T1 cocktail was added for 20 min at 37°, and then samples were lysed for total RNA extraction using Zymo Quick-RNA viral kits according to instructions. RNA was reverse transcribed with random primers and ZIKV genomes were quantified with qPCR. Results were normalized to ZIKV + PBS without RNAse condition. Three independent experiments with duplicate wells for each condition were done.

## Results

### Zika Virus Productively Infects Primary Vaginal, Ectocervical, and Endocervical Epithelial Cells

**Figure 1:**
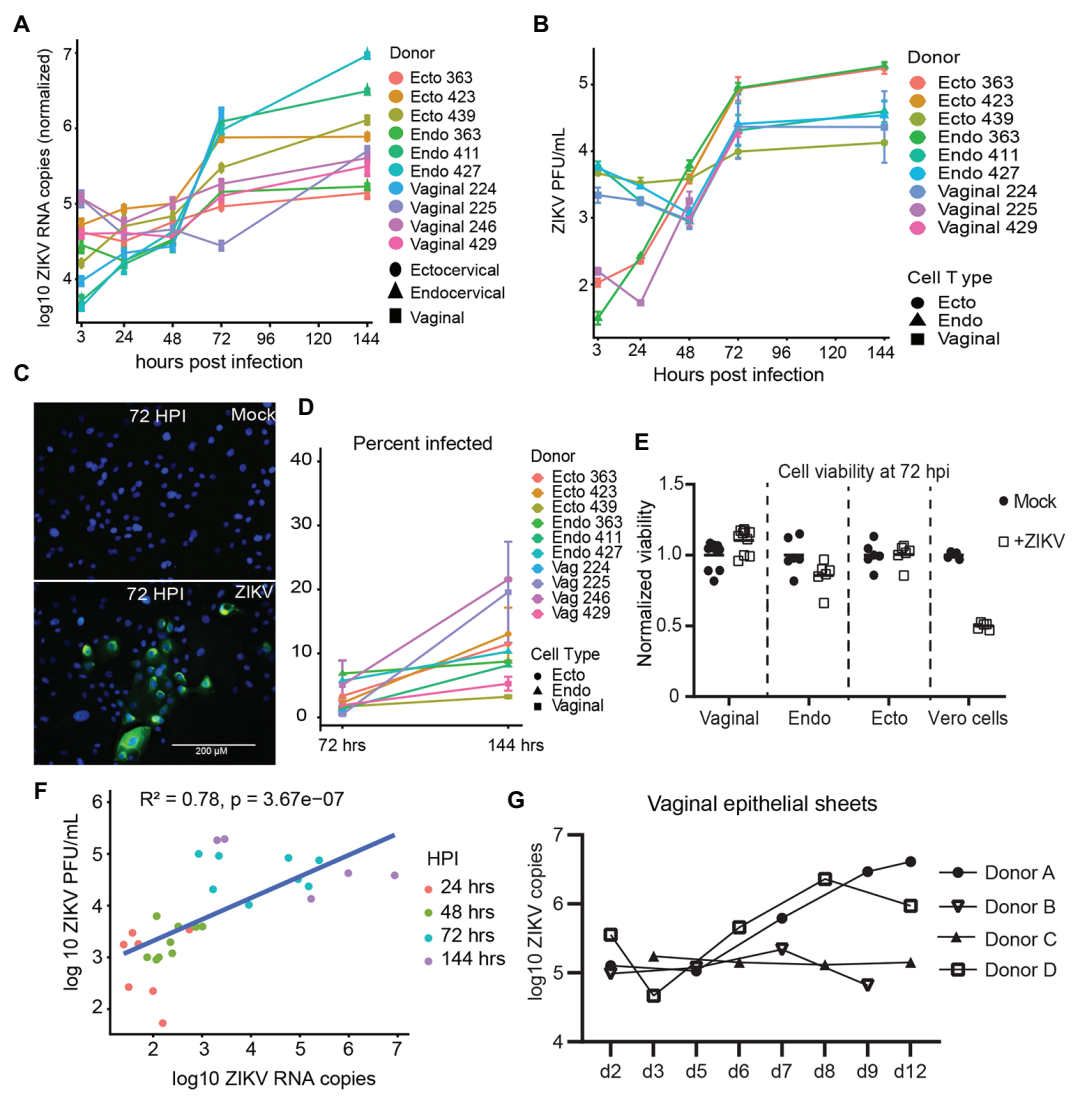
Kinetics of Zika virus (ZIKV) replication in female genital tract epithelial cells and tissue. Epithelial cells were exposed to ZIKV at a multiplicity of infection (MOI) of 1 for 1.5 h, washed, and then cultured. At the indicated time points, cells were processed for quantification of ZIKV infection by multiple methods. **(A)** RNA from infected cells was reversed transcribed, and copies of ZIKV virus genome and cellular housekeeper RPP30 were quantified by droplet digital PCR (ddPCR). Y-axis indicates the log10 copy number of ZIKV genomes detected in 200 ng of input RNA, normalized to RPP30 levels. Each line is the average results for duplicate wells, error bars are standard deviation, and each color is a separate experiment. **(B)** Supernatants from infected cells were added to Vero cells for plaque assays as described in Materials and Methods section. Plaque-forming units (PFU) per ml of supernatant averaged over three separate wells are plotted; error bars are standard deviation. **(C)** Staining for ZIKV envelope protein. At 72 hpi, cells were fixed in 4% paraformaldehyde, permeabilized, stained with anti-flavivirus envelope 4G2, and counterstained with DAPI. One representative of nine separate experiments is shown, **(D)** Kinetics of the percent of ZIKV-infected cells. At least 500 DAPI+ cells per well were analyzed for ZIKV envelope E protein expression to calculate the percentage of infected cells. Each point is the average of two separate wells, error bars are standard deviation, and each line is a separate experiment. **(E)** Cell viability was assessed with the Viral ToxGlo assay. Fold difference from the mean of values for mock infected cells is plotted. Vero cells were infected with ZIKV at an MOI of 0.05 and assessed for cytotoxicity at 72 hpi as a positive control. Each point is a separate well. **(F)** Correlation between ZIKV RNA copies measured by ddPCR and ZIKV PFUs as determined by plaque assay. Data generated from at 48, 72, and 144 hpi time points were pooled for the regression. **(G)** Epithelial sheets from vaginal tissue were exposed to 500,000 PFU of ZIKV overnight, extensively washed, and cultured for the indicated times. At harvest, total tissue RNA was extracted, and ZIKV genomes quantified by ddPCR and normalized to RPP30 levels. Points are the average of two or three independent wells per experiment/donor.

We established primary, oncogene-untransformed, epithelial cell lines from vaginal, ectocervical, and endocervical human tissues (Hladik et al., 2007, 2015). These cells were infected with Zika VR-1848, a strain isolated from the placenta of a patient in Honduras, at a MOI of 1. We tested at least three cell lines derived from different individuals for each anatomical site. Cultures were harvested at 3, 24, 48, 72, and 144 hpi to establish the timeline of viral replication. Genome copies of ZIKV in cells were measured with RT-ddPCR and normalized to the housekeeper gene RPP30. Productive infection was evident in all cell lines, with an average increase of 1.47 log10 copies of RNA (range 0.51–3.33 log10 copies) between 3 hpi (considered input virus level) and 144 hpi. While viral levels compared to input dropped or stayed relatively steady until 48 hpi, in all but one sample they rapidly rose between 48 and 72 hpi (Figure 1A), indicating that viral replication occurs most rapidly beyond 48 hpi in these cells. Beyond 72 hpi, viral levels increased at a slower rate. Infectious virus was present in the supernatant of infected epithelial cells at between 4.14 log10 and 5.13 log10 PFUs per ml at 144 hpi (Figure 1B). As additional confirmation of productive infection, cultures were fixed, stained for expression of viral envelope protein E (Figure 1C), and counted to determine the percent of cells infected at 72 and 144 hpi. We detected clusters of cells that stained for high levels of viral proteins in the cytoplasm (Figure 1C). The percent of infected cells in cultures increased between 72 and 144 hpi (Figure 1D). We did not see evidence for a difference in viral susceptibility or replication between vaginal, ectocervical, and endocervical cells. However, we did see donor-to-donor variation in peak levels of RNA. The cell line with the highest RNA viral load at 144 hpi (endocervical 427) had a 2146.0-fold increase of viral genome copies compared to loads at 3 hpi, while the least susceptible cell line (ectocervical 363) had a 3.3-fold increase (median 8.36-fold increase in all cell lines). Despite clear productive infection of the epithelial cells, we did not observe any cytotoxic effects of ZIKV, either visually or using a viability assay. This contrasted to Vero cells, which exhibit a pronounced cytopathic effect upon ZIKV challenge (Figure 1E).

We confirmed replication of ZIKV in *ex-vivo* vaginal tissue explants from four individual donors. Tissues were processed into epithelial sheets as previously described (Hladik et al., 2007, 2015), and infected with 500,000 PFU of ZIKV overnight. Sheets were extensively washed, preserving emigrated cells, which were added back, and cultured for the indicated periods. Because RNA levels of ZIKV correlated well with production of infectious virus in culture supernatants (Figure 1F), we chose RNA assays as the primary readout of infection in the rest of our experiments. In three of the four tissue samples, ZIKV established productive infection, as indicated by an increase in viral copy number in epithelial sheets after day 3, while in one donor (Donor C) viral levels remained mostly stable over 12 days of culture (Figure 1G, donor C). Again, we observed donor-to-donor variation in replication of ZIKV, with a peak increase of 32-fold in one donor compared to no increase in another (average peak replication of 10.4-fold between days 7 and 12 after infection). We made a considerable effort to detect ZIKV infection in intraepithelial leukocytes, particularly in Langerhans cells (LC) and T cells emigrating from the tissues, as we have documented during HIV infection (Hladik et al., 2007; Ballweber et al., 2011) but were not able to find evidence of measurable ZIKV infection in these cells. Taken together, these results show that viral replication occurs in various types of genital epithelial cells.

### Zika Virus Induces an Antiviral Immune Response in Genital Epithelial Cells

**Figure 2:**
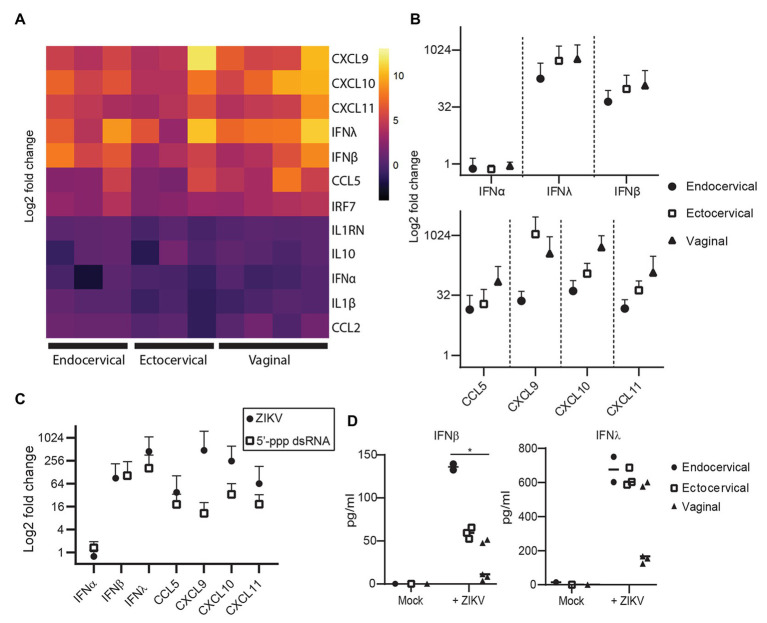
Immune response of genital epithelial cells to ZIVK infection. **(A)** RNA from cells infected with ZIKV at an MOI of 1 for 72 h was analyzed for the indicated genes by quantitative PCR (qPCR). Heatmap of log2 fold regulation of each analyzed gene compared to mock infected control cells, averaged from two independent wells. Cell type is indicated on the *x*-axis; each column is a separate cell line. **(B)** Numerical representation of the data shown in panel **(A)** averaged for each tissue type. Error bars indicate one standard deviation. **(C)** Three epithelial cell lines (one for each tissue type) were treated with 10 μg/ml of the RIG-I agonist 5'ppp-dsRNA complexed with LyoVec (*Invivo*Gen), or infected with ZIKV at an MOI of 1 for 24 h. RNA was isolated and gene expression of the indicated cytokines was measured by qPCR. Plotted is the mean response of all epithelial cell lines to ZIKV infection at 72 hpi compared to the mean response to 5'ppp-dsRNA. **(D)** Supernatants from cell cultures in **(A)** were analyzed for IFNα, IFNβ, and IFNλ using a Meso Scale Discovery assay. IFNα was below the limit of detection in all samples and is not shown. Concentration of IFNβ and IFNλ in ZIKV-infected and mock-control cell culture supernatants are shown, each point is the average of two independent wells, and at least two cell lines from each tissue type were tested. Horizontal bars indicate mean concentrations. Differences between cell types were tested by one-way ANOVA with Tukey’s test. ^*^ *p* < 0.05.

To evaluate the immune response of primary epithelial cells to infection with ZIKV, we examined the mRNA expression levels of 13 anti-viral genes at 72 hpi, when viral replication is robust. Of the type I interferons, IFNβ (IFNB1) but not IFNα (IFNA1) was upregulated compared to mock controls (Figures 2A,B). As has been reported by others (Khan et al., 2016, 2019), the type III interferon IFNλ2 (IFNL2 or IL-28A) was also strongly upregulated. We observed very strong induction of CCL5 and the related CXCL family members 9, 10, and 11 (Figures 2A,B). We did not see clear evidence of a difference between the immune responses of epithelial cells derived from vaginal, ectocervical, or endocervical tissues at the mRNA level, though there was a trend for vaginal tissue-derived cells to respond most robustly (Figure 2B). ZIKV specifically degrades STAT2 to impair type I interferon signaling (Grant et al., 2016). To determine if the observed immune response to ZIKV occurs through bypassing STAT2 signaling, we treated epithelial cells with 5' triphosphate (ppp)-dsRNA, a RIG-I agonist which initiates immunity through STAT2 independent pathways. Interferon responses to 5'ppp-dsRNA and ZIKV were similar, with no effect on IFNα and strong upregulation of both IFNβ and IFNλ, indicating that ZIKV induces a maximal IFNβ/λ transcriptional response in these cells (Figure 2C). Chemokines were upregulated to a greater extent in ZIKV infected cells than in 5'ppp-dsRNA treated cells (Figure 2C). This implies that ZIKV infection induces a vigorous chemokine response through multiple innate immune pathways.

Because some reports have shown that cells can upregulate interferons at the transcriptional level but do not actually secrete these interferons (Quicke et al., 2016; Bowen et al., 2017), we surveyed the secretion of IFNα, IFNβ, and IFNλ protein in the supernatants of a subset of infected cells. IFNα was not detected in these supernatants (data not shown, all samples below the limit of detection). However, we did detect a significant difference in the levels of IFNβ secreted in supernatants by tissue type, with endocervical cells secreting the most (mean 136.0 pg/ml), followed by ectocervical (59.2 pg/ml) and vaginal cells (49.5 pg/ml). The trend for IFNλ was the same, though the difference was not significant (Figure 2D). This was opposite to the trend for IFNβ and IFNλ at the transcriptional level (Figure 2B). This suggests some decoupling of the transcriptional and translational regulation of these interferons in genital epithelial cells, with endocervical cells well poised to secrete IFNβ/λ in response to ascending viral infection even if their transcriptional activity of these interferons is lower than in ectocervical and vaginal cells.

### Extracellular Vesicles From Semen Inhibit Zika Virus Infection

**Figure 3:**
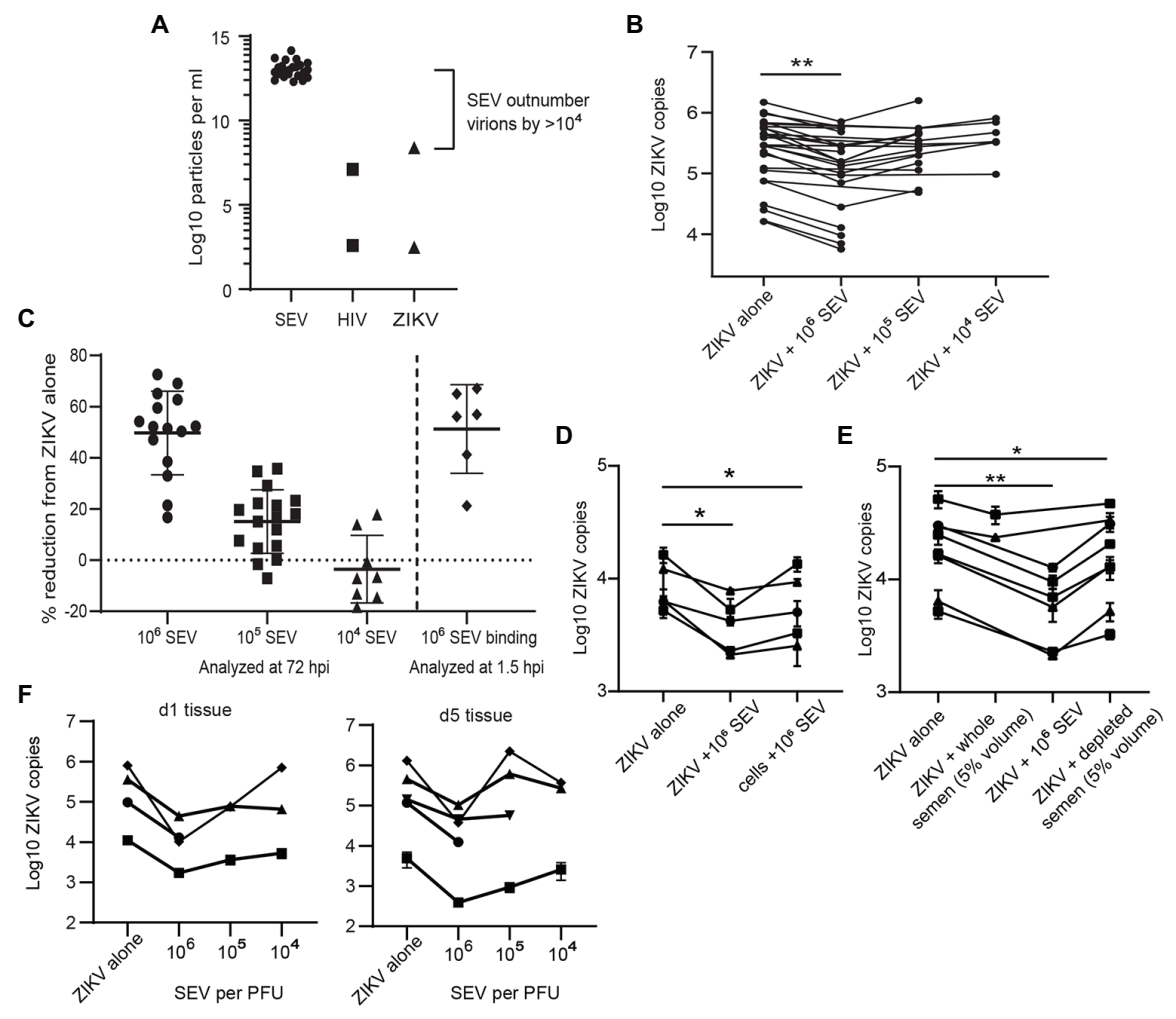
Seminal extracellular vesicles inhibit ZIKV binding and infection. **(A)** Comparison of the average yield of SEV per ml of semen (*n* = 23 donors) and reported viral RNA loads for ZIKV (Mead et al., 2018) and HIV (Gupta et al., 1997) viruses. Points for HIV and ZIKV represent high and low estimates of viral loads in semen. **(B)** ZIKV was incubated with 10^6^, 10^5^, or 10^4^ SEV per PFU of virus for 1 h at 37° prior to infection of genital epithelial cells at a MOI of 1. After 1.5 h at 37°, the inoculum was removed and cells were cultured for 72 h. Log copies of ZIKV RNA quantified by ddPCR and normalized to the housekeeper gene RPP30 are plotted, averaged over duplicate wells. Each line represents a different cell line. Decrease in ZIKV genomes in the 10^6^ SEV condition is significant by mixed-effects analysis with Dunnett’s multiple comparisons test. ^**^ *p* < 0.01. **(C)** Quantification of inhibition of infection at 72 and 1.5 h post-infection. For 72 hpi, data from **B** is plotted as percent reduction of ZIKV genome copy number, measured by ddPCR, compared to cells exposed to ZIKV alone. For “SEV binding,” genital epithelial cells simultaneously exposed to ZIKV and 10^6^ SEV per PFU were lysed at 1.5 hpi. Each point is a separate cell line, and horizontal bars indicate means. ^**^ *p* < 0.05. **(D)** Comparison of pre-incubation of either ZIKV or epithelial cells with SEV on viral infection. SEVs were pre-incubated with ZIKV (ratio:10^6^ SEV per PFU) or with cells (ratio: 10^6^ SEV per cell) for 1 h at 37°, then virus was added to cells for 1.5 h. Cells were washed and cell-associated ZIKV genomes were quantified by ddPCR. Each line represents the average of two technical replicates from different epithelial cell lines. Significance by one-way ANOVA with repeated measures, with Dunnett’s multiple comparisons test. ^*^ *p* < 0.05. **(E)** ZIKV was pre-incubated with whole semen containing the equivalent of 10^6^ SEV per PFU (approximately 5% volume), 10^6^ isolate SEV per PFU, or with 5% volume of SEV-depleted seminal plasma. Samples were added to cells for 1.5 h, then ZIKV genomes quantified by ddPCR. Each line represents the average of two technical replicates from different epithelial cell lines significance as in **D**. ^**^ *p* < 0.01. **(F)** *Ex-vivo* vaginal tissues were infected overnight with 500,000 PFU of ZIKV alone, or ZIKV pre-incubated for 1 h at 37° with 10^6^, 10^5^, or 10^4^ SEV per PFU of virus. Tissues were washed extensively and then lysed for RNA isolation and quantification of ZIKV genomes by ddPCR immediately (d1 following addition of virus) or were cultured for an additional 4 days prior to RNA isolation. Each symbol denotes the average of two wells, error bars are standard deviation, and each line is a different tissue donor.

Because sexually transmitted ZIKV is delivered in the presence of semen, we sought to determine if components of semen influenced ZIKV infection. Isolated SEV inhibit HIV infection (Madison et al., 2014, 2015; our unpublished data), and were more recently reported to also inhibit ZIKV virus infection (Müller et al., 2018). Following purification of SEV from semen, we detect an average of 9.85 × 10^12^ SEV per ml of ejaculate (*n* = 23 samples; Figure 3A). The range of ZIKV viral load reported in semen is 10^2.5^–10^8.4^ copies per ml, with >10^7^ copies considered a high viral load (Mead et al., 2018; Medina et al., 2019). This is close to highest HIV viral loads detected in semen, which is also around 10^7^ copies, with a more typical range of 10^2^–10^6^ copies per ml (Gupta et al., 1997; Kalichman et al., 2001; Pilcher et al., 2007). This means that even with the highest viral loads ever measured, SEV outnumber ZIKV virions in semen by at least 10^4^, and more typically by 10^6^–10^8^ vesicles per virion (Figure 3A). Therefore, to mimic physiological conditions, we incubated ZIKV virions with SEV isolated and pooled from five individuals at 10^4^, 10^5^, and 10^6^ SEV per PFU prior to infecting genital epithelial cells. Infection rates were determined by assaying RNA viral load at 72 hpi. SEV significantly inhibited ZIKV infection in genital epithelial cells in a dose-responsive manner (Figure 3B). Viral RNA copies were reduced by an average of 49.7% for the 10^6^ SEV/PFU and 15.1% for the 10^5^ SEV/PFU ratio (Figure 3C). The inhibition of infection was lost at the lower ratio of 10^4^ SEV per PFU. To clarify whether SEV blocked initial steps of infection, or viral replication post entry, cells infected as above were washed, lysed, and assayed for ZIKV genomes at 1.5 hpi, just following the initial incubation period but before active viral replication. We found that even at 1.5 hpi, SEV impaired binding or entry of ZIKV to cells with the same efficiency observed at 72 hpi (51.3% for 1.5 hpi compared to 49.7% at 72 hpi; Figure 3C), indicating that SEV interfere with initial binding or entry. Though pre-incubating SEV and virions is the most physiological experimental setup, we also tried pre-incubating cells with SEV for 1 h and then washing the SEV off the cells prior to adding ZIKV. We found that this setup showed a more limited impairment of ZIKV infection, though levels of ZIKV were still lower than in the ZIKV alone condition (Figure 3D). This suggests that the antiviral activity of SEV is most potent when SEV and virions are together at the time of infection.

We also tested the effects of whole semen containing an equivalent of 10^6^ SEV per PFU (5% semen by volume), and vesicle-depleted semen. Since seminal plasma can be cytotoxic to cell cultures (Allen and Roberts, 1986), we tested binding of ZIKV to cells in the presence of whole semen or SEV-depleted semen at 1.5 hpi, when the effect of SEV was already apparent but the cytotoxic effect would not confound results. We found that whole semen containing SEV impaired ZIKV binding to cells (22.9% inhibition, not significant), though not to the same extent as isolated SEV (61% inhibition, significant; Figure 3E). SEV-depleted seminal plasma had a much smaller effect on ZIKV binding than the SEV fraction (18.8% reduction in ZIKV levels for seminal plasma; Figure 3E).

In addition to examining the effect of SEV on ZIKV infection in *in vitro*-cultured genital epithelial cells, we repeated these experiments in *ex-vivo* vaginal tissues. Epithelial sheets were infected with 500,000 PFU of ZIKV alone or in the presence of 10^6^, 10^5^, or 10^4^ SEV per PFU of virus overnight, and then washed extensively and assayed for ZIKV RNA immediately (d1) or cultured for an additional 4 days before analysis (d5). Epithelial sheets derived from *ex-vivo* tissue have inherent variation and had different levels of virus detected at d1, 24 h after exposure. Nonetheless, the presence of SEV in ZIKV inoculum restricted infection of epithelial sheets even more potently than in isolated epithelial cells when compared to virus alone conditions. At the highest ratio of 10^6^ SEV/PFU, SEV inhibited infection at d1 by an average of 89.3% (range 84.4–98.7%) and this inhibition was still apparent at d5 post-infection, with an average 84.6% reduction in ZIKV genome copies (range 67.5–97.1%; Figure 3F). At the d1 time point, 10^5^ and 10^4^ SEV/PFU were also inhibitory (average 78.5 and 48.2%), but by d5 in some tissues ZIKV infection had overcome the effect of lower amounts of SEV (Figure 3F). Taken together, these data indicate that at physiological ratios of SEV to ZIKV virions, SEV exert a potent inhibitory effect on ZIKV that prevents productive infection. Only in cases where viral load is very high, lowering the ratio of SEV to virions, is ZIKV able to overcome this inhibitory effect and establish productive infection.

### Inhibition of Zika Virus Binding by Extracellular Vesicles in Semen Is Mediated by Lipids

**Figure 4:**
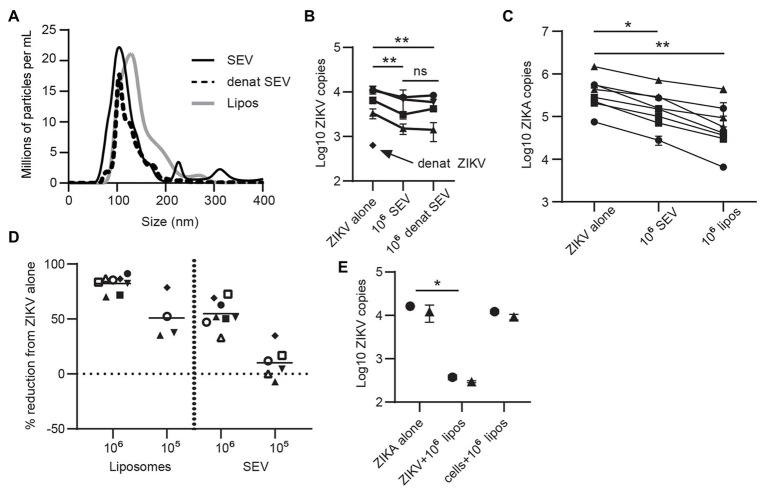
Lipid content of SEV blocks ZIKV infection. **(A)** The protein content of SEV was denatured by heating at 95° for 8 min. Nanoparticle tracking analysis (NTA) profile comparing heat-treated and untreated SEV showing that the size profiles do not change. Size profiles of liposomes (see part C) are also plotted. **(B)** Equal amounts of heat-treated or untreated SEV were incubated with ZIKV virions at a 10^6^ SEV:PFU, then added to epithelial cell lines for 1.5 h before washing and assessing ZIKV binding by ddPCR. As a control, ZIKV virions were also heated at 95° for 8 min. Each line is a separate cell line, points are mean of two replicate wells and error bars are standard deviation. Significance by one-way ANOVA with Tukey’s multiple comparisons test. ^**^p > 0.01. **(C)** ZIKV was pre-incubated with 10^6^ SEV or 10^6^ liposomes for 1 h prior to infecting genital epithelial cells. Seventy-two hours post-infection cells were lysed and ZIKV genomes quantified by ddPCR. Each line is the average of duplicate wells from a separate experiment. Reduction in ZIKV genomes was significant by one-way ANOVA with Dunnett’s multiple comparisons test. ^*^p < 0.05, and ^**^p < 0.005. **(D)** The data from **C**, and additional conditions with 10^5^ SEV or liposomes, are plotted as percent reduction from ZIKV alone. Each symbol represents the average of two technical wells from a different cell line. The horizontal lines are the mean for each condition. **(E)** Comparison of pre-incubation of either ZIKV or epithelial cells with liposomes on viral infection. Liposomes were pre-incubated with ZIKV (ratio:10^6^ liposome per PFU) or with cells (same amount of liposomes) for 1 h at 37°, then virus was added to cells for 1.5 h. Cells were washed and cell-associated ZIKV genomes quantified by ddPCR. Each symbol represents the average of two technical replicates from different epithelial cell lines. Significance by one-way ANOVA with Dunnett’s multiple comparisons test. ^*^p < 0.05.

Extracellular vesicles contain three primary classes of biomolecules: lipids, proteins, and nucleic acids. To determine which cargo plays a role in inhibiting ZIKV, we heat-treated SEV to denature their protein cargo. This treatment did not alter the size profile of SEV (Figure 4A), indicating that the vesicles remained intact.

Denaturing ZIKV strongly impaired infection, as expected. Denatured SEV inhibited ZIKV infection to the same extent as untreated SEV (mean 44.5% reduction compared to 41.1% reduction; Figure 4B). This implied that the lipid components of SEV could have been responsible for their inhibitory effect. To test this, we generated liposomes of the same size and lipid content as SEV (Brouwers et al., 2013; Table 2 in Materials and Methods section; Figure 4A). These liposomes are also entirely free of nucleic acids. Liposomes were pre-incubated with ZIKV virions at 10^6^ and 10^5^ liposomes per PFU, and then both were added to the cells for 1.5 h, as we did for SEV. Liposomes impaired ZIKV binding and infection in genital epithelial cells slightly more potently than SEV but in a similar dose range, reducing viral RNA copies by 82.3% at 10^6^ and 51.0% at 10^5^ (Figures 4C,D). We also tested adding liposomes to cells before infecting with ZIKV. ZIKV infection was inhibited much less when liposomes were added to cells rather than pre-incubating them first with the virions (97.6% inhibition for ZIKV + liposomes, compared to 24.6% inhibition for cells + liposomes; Figure 4E). Because liposomes lack proteins and nucleic acids, these results show that the lipid fraction of SEV is primarily responsible for their interaction with ZIKV virions and inhibition of ZIKV infection.

### Extracellular Vesicles and Liposomes Do Not Block Phosphatidylserine Receptors, But Cause a Loss in Zika Virus Membrane Integrity

**Figure 5:**
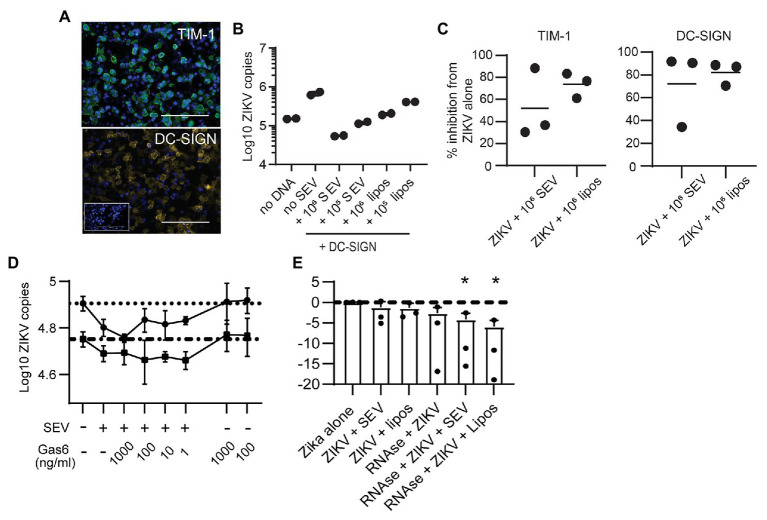
Seminal extracellular vesicles and liposomes inhibit ZIKV by causing loss of virion membrane integrity. **(A)** HEK293 cells were transfected to express the DC-SIGN or TIM-1 receptors. Twenty-four hours post transfection, cells were stained with antibodies against TIM-1 (top panel, green) or DC-SIGN (bottom panel, yellow) to visualize expression of receptors. DAPI staining visualizes the cell nucleus (blue). Mock-transfected cells incubated with anti-DC-SIGN are shown in the inset for the DC-SIGN panel. Scale bar is 200 μm for all images (imaged at 20x magnification). **(B)** ZIKV was incubated alone or with 10^6^ SEV or liposomes per PFU for 1 h prior to infecting DC-SIGN transfected cells at an MOI of 1 for 1.5 h. Inoculum was removed and cells were cultured for 24 h, then cultures were lysed. Log copies of ZIKV RNA quantified by ddPCR and normalized to the housekeeper gene RPP30 are plotted. Each symbol is from one well. Plot is a representative result from two separate experiments. **(C)** HEK-293 cells transfected to express DC-SIGN or TIM-1 were infected as in B, and the percent inhibition from the ZIKV alone condition is plotted. Each symbol is the average of two duplicate wells from two separate experiments and the line indicates the mean **(D)** ZIKV was incubated alone or with 10^5^ SEV per PFU for 1 h, prior to infecting cells. During incubation with viral inoculum, recombinant Gas6 protein was added to the culture at the indicated concentrations. After 1.5 h, viral inoculum was removed and cells were lysed to quantify ZIKV genomes with ddPCR. Each line represents the average of two technical replicates from two different epithelial cell lines, done in separate experiments. Dotted lines show the copy numbers obtained in the ZIKV alone condition for each cell line. **(E)** ZIKV virions were incubated alone or with 10^6^ SEV or liposomes for 1 h at 37°. One microliter of an RNAse A/T1 cocktail was added for 20 min at 37°, and then samples were lysed for total RNA extraction. ZIKV genomes were quantified with qPCR. Log10 copies of ZIKV virus genome normalized to the virus alone condition are plotted; the line indicates ZIKV alone. Results from three independent experiments are shown as dots; bars are mean and lines are standard deviation. ^*^ *p* < 0.007 by one-way ANOVA with Tukey’s multiple comparisons test.

Flaviviruses can use many different cellular receptors for entry. Among the most described are receptors such as AXL and TIM-1 that bind phosphatidylserine (PS) in the viral envelope, either directly or *via* Gas6 or other bridging co-receptors (Hamel et al., 2015). Like ZIKV, exosomes contain exposed PS molecules (Colombo et al., 2014). Thus, we reasoned SEV could inhibit ZIKV infection either by blocking PS receptors on cells or by sequestering the Gas6 co-receptor. If this were true, SEV and liposomes should not block ZIKV entry *via* a receptor which binds glycoproteins rather than PS. To test this experimentally, we transfected HEK293 cells with either TIM-1, a PS-binding receptor, or DC-SIGN, a receptor which binds glycoproteins (Figure 5A). SEV inhibited ZIKV infection strongly and to nearly identical extents in cells expressing either DC-SIGN (Figures 5B,C; mean inhibition by 10^6^ SEV/virion 63.2%) or TIM-1 (Figure 5C; mean inhibition by 10^6^ SEV/virion 59.4%). 10^6^ liposomes/virion also had strong inhibitory effects (78.9% for DC-SIGN and 69.0% for TIM-1).

To assess involvement of Gas6, we infected two genital epithelial cell lines with ZIKV alone, or with ZIKV pre-incubated for 1 h with SEV, and added Gas6 protein back to cultures at the time of infection. Typical plasma concentrations of Gas6 are 10–100 ng/ml, and 10% serum contains a sufficient amount of Gas6 to promote infection (Ekman et al., 2010; Morizono and Chen, 2014), so our highest dose of 1,000 ng/ml is at least 100 times more Gas6 than should be sufficient for infection. Adding Gas6 at any dose did not reverse inhibition of infection by SEV, nor did it enhance infection of ZIKV alone (Figure 5D), indicating that SEV do not impair ZIKV infection by sequestering Gas6. Taken together, these results show that SEV block ZIKV infection independently of the cellular viral receptor or the bridging co-receptor.

These findings led us to hypothesize that SEV and liposomes interact directly with ZIKV to impair infection, possibly by fusing with virions and causing loss of viral integrity. To test whether the ZIKV genome remained protected during interactions with SEV or liposomes, we treated ZIKV with SEV or liposomes, then added RNAse and quantified the remaining genome copies of ZIKV. We found that both SEV and liposomes caused a loss in ZIKV genome detection in the presence of RNAse (mean 0.057 fold ZIKV copies with SEV + RNAse and 0.017 fold ZIKV copies with liposomes+RNAse, normalized to 1 for ZIKV alone; Figure 5E). The loss of ZIKV genome integrity in the presence of SEV or liposomes suggests a direct interaction between SEV or liposomes and virions that results in a virocidal effect.

## Discussion

Our findings show that ZIKV productively infects primary epithelial cells of the female genital tract, inducing an antiviral immune response, yet continues to replicate without cytotoxic effect. Early in the epidemic, there was much concern regarding the sexual transmission of ZIKV due to the detection of high and persistent viral loads in the semen of men (Mansuy et al., 2016). However, sexual transmission of ZIKV has turned out to be relatively rare, indicating that most of the ZIKV RNA detected in semen is from defective virus and/or that as yet unknown biological factors oppose efficient ZIKV transmission. Long-term detection of ZIKV in semen and considerable damage to genital tissues in men (Ma et al., 2016; Siemann et al., 2017) suggest that the male genital tract is a site of persistent replication of competent virus. This virus likely is present in ejaculated semen (Govero et al., 2016; Ma et al., 2016; Joguet et al., 2017; Matusali et al., 2018; Robinson et al., 2018; Stassen et al., 2018), arguing against the defective virus explanation. In addition, primate and mouse studies both report successful transmission of ZIKV *via* semen (Uraki et al., 2017; Gurung et al., 2020). On the other hand, our findings here demonstrate that extracellular vesicles from semen are potent inhibitors of ZIKV infection at physiological ratios, providing a plausible reason why sexual transmission of ZIKV is a rare event. Our data are in agreement with another recent report (Müller et al., 2018). We further show that this effect can be mimicked by naked liposomes and that the mechanism of this effect is not by blocking cellular receptors or co-receptors, but rather *via* an interaction between SEV and virions.

We confirmed that ZIKV infects and replicates in epithelial cell lines from female genital tract tissues. We tested a minimum of three primary cell lines from independent donors derived from vaginal, ectocervical, or endocervical tissues, and observed no differences between viral replication dynamics or susceptibility to infection between these anatomical sites (Figures 1A,B,D). Vaginal explant tissues were also productively infected with ZIKV, though they reached peak viral replication more slowly than isolated cell lines (Figure 1G). In interferon-competent mice, vaginally-delivered ZIKV replicates in the genital tract without causing systemic infection (Yockey et al., 2016). This mirrors observations in human populations where detection of ZIKV in vaginal secretions is rare but occurs in a subset of women, potentially those infected sexually (Prisant et al., 2016; Visseaux et al., 2016; Paz-Bailey et al., 2018). Given the observed robust replication of ZIKV in cells of the female genital tract, it is reasonable to speculate that sexual transmission of ZIKV increases the risk of fetal infection and subsequent negative outcomes in pregnant women.

We did not observe cytopathic effects of ZIKV infection in our epithelial cell lines during the observation time (Figure 1E). A lack of cell death has also been observed in some testicular cell lines, in dendritic cells, and in placental macrophages (Quicke et al., 2016; Vielle et al., 2016; Mlera and Bloom, 2019). This is likely due to an intact innate antiviral immune response characterized by high levels of IFNβ and IFNλ (Figure 2), which might limit infection and cell death. Induction of interferon and interferon-stimulated genes by ZIKV, and control of viral replication by the IFN pathway, has been well-documented, including in vaginally-introduced infections (Hamel et al., 2015; Bayer et al., 2016; Lazear et al., 2016; Caine et al., 2019; Khan et al., 2019). IFNλ is critical to antiviral activity in the female genital tract and also plays an important role in protecting primary placental cells from ZIKV infection (Iversen et al., 2010; Bayer et al., 2016). In contrast to experiments in ZIKV-infected placental macrophages (Hofbauer cells) and monocyte-derived dendritic cells, where IFNβ was induced at the transcriptional level but not secreted into supernatants (Quicke et al., 2016; Bowen et al., 2017), we did detect secretion of IFNβ from infected epithelial cells (Figure 2D). Interestingly, we observed that transcription and translation of IFNβ and IFNλ might be differentially regulated in cells derived from different parts of the female genital tract. Though mRNA was induced at similar levels in cells from all tissues, IFNβ was secreted from endocervical cells at much higher levels than from ectocervical or vaginal cells (Figures 2B,D). Enhanced expression of viral RNA sensors and responsiveness to IFN in the endocervix, as compared to ectocervix or vagina, has been reported in mice (Khan et al., 2019). The endocervix is typically considered sterile, as opposed to the ectocervix and vagina, which are exposed to a resident microbiome and to exogenous bacteria and viruses *via* sexual intercourse and hygienic practices. It makes sense that the endocervix has heightened immune sensing and responsiveness, compared to the vagina and ectocervix, to combat ascending infectious agents.

We treated a subset of epithelial cells with a RIG-I agonist to determine a maximal RIG-I induced immune response in these cells to compare to the response to ZIKV infection. ZIKV subverts type I IFN signaling by degrading STAT2, but RIG-I signaling proceeds through STAT2 independent pathways (Kawai and Akira, 2008). Though there is some evidence that ZIKV also antagonizes RIG-I signaling, this pathway senses ZIKV infection early and is very potent at restricting ZIKV (Grant et al., 2016; Bowen et al., 2017; Wu et al., 2017; Hu et al., 2019). At the peak of viral replication in genital epithelial cells (72 hpi), IFNβ and IFNλ were upregulated to the same extent as following RIG-I ligand stimulation (Figure 2C), demonstrating that ZIKV induces a maximal interferon response in these cells. Similarly to infections in skin fibroblasts (Hamel et al., 2015), ZIKV infection in genital tract epithelial cells strongly enhanced expression of CXCL9, CXCL10, CXCL11, and CCL5 to an even greater extent than treatment with the RIG-I agonist, indicating that chemokines are likely induced through multiple signaling pathways in these cells (Figure 2D). Given the relatively low percent of cells that stained positive for ZIKV envelope protein (Figures 1C,D), the strong upregulation of chemokines implies that cells that are not productively infected are also responding to viral PAMPs or IFNs.

We observed strong inhibition of ZIKV binding to and infection of cells when virus was pre-incubated with SEV, as would be the condition during sexual transmission of ZIKV when virus is delivered in ejaculates (Figures 3B–F). The inhibitory effect of semen on ZIKV is linked to the EV fraction, as using an equivalent volume of SEV-depleted seminal plasma resulted in much reduced inhibition (Figure 3E). This was also recently reported by Müller et al. (2018). We also tested whole semen, and found it was inhibitory to ZIKV, but not to the extent expected since the volume we tested has the same SEV concentration as isolated SEV (Figure 3E). This could be because semen is a very viscous fluid. Even though we allow it to liquefy before processing, there is a very high protein concentration, including fibril-forming proteins, while isolated SEV contain only about 10% of the protein content of whole semen. We saw that the interaction between SEV and virions is an important part of how SEV inhibit ZIKV, so in whole semen, which is much more viscous that isolated SEV, virions and SEV may not be able to diffuse enough to contact each other and cause ZIKV inactivation to the extent we would expect by SEV concentration alone. *In vivo*, the situation would be different. Multiple sites in the male genital tract can be infected with ZIKV (Stassen et al., 2018), meaning virions are likely generated alongside SEV throughout the entire male genital tract and before coagulation of semen upon ejaculation. This could inactivate virions before they even leave the male body. Secondly, in the female genital tract, seminal fluid becomes less viscous over time and *via* dilution with vaginal fluids, likely allowing greater interaction between virions and SEV and inactivation of ZIKV. Further studies in semen samples from infected men or in animal models are warranted to determine the extent of whole semen inhibition of Zika virus both before and after ejaculation.

We extended these findings to show that the inhibitory property of SEV is most likely linked to their lipid composition. Heat-treatment of SEV to denature proteins did not prevent their ability to block ZIKV infection, implying that functional proteins are not required for this effect (Figure 4B). Liposomes engineered to the same lipid content and size as SEV, but containing no protein or RNA cargo, were even more efficient than SEV at preventing ZIKV binding and infection of cells, presumably due to greater lipid accessibility when cellular membrane proteins are not present, as they are in SEV (Figures 4C,D). We based the lipid content on published studies of bulk SEV lipid composition (Brouwers et al., 2013). Though we expect our liposomes mimic the lipid composition of SEV, it is possible that individual SEV may vary in lipid content, and that a particular subset may be more or less inhibitory *in vivo*. Given that the lipid content of SEV appears to be responsible for their inhibitory effect, the role of individual lipid species may be important to investigate in further studies.

Studies report that EVs carry exposed PS (Colombo et al., 2014), as do our liposomes. We considered that SEV might be blocking PS receptors on cells, explaining how they prevent viral binding. If this were the case, pre-treatment of cells with SEV or liposomes should result in the same or greater blocking of viral binding. However, pre-incubating SEV with cells was less effective than pre-incubating SEV directly with virions (Figure 3D). This effect was even more pronounced with naked liposomes, which would presumably express even more exposed PS (Figure 4D). Additionally, SEV and liposomes were able to block infection in cells expressing DC-SIGN, a lectin receptor that binds glycan residues on viral proteins (Figures 5B,C). Though we cannot rule out that DC-SIGN could also bind SEV, it is very unlikely that naked liposomes would be able to block DC-SIGN directly. We also considered that SEV could be binding and sequestering Gas6, a bridging co-receptor which binds PS and the primary ZIKV receptor AXL along with other TAM family receptors (Hamel et al., 2015). However, adding back high concentrations of Gas6 during infection did not restore high rates of viral binding in the presence of SEV or liposomes (Figure 5D). Together, these results suggested that SEV and liposomes interact with ZIKV virions, rather than with cells, to prevent binding to cells. Incubating ZIKV with SEV or liposomes caused sensitivity to RNAse degradation of ZIKV (Figure 5E). This suggests that SEV or liposomes induce premature fusion or uncoating of virions in solution, preventing their ability to effectively bind to susceptible cells. We presume the mechanism of this effect is *via* electrostatic interactions, a hypothesis we will investigate further in future work.

We observed a concordance between the ratios of SEV and infectious virions that are inhibitory in our *in vitro* experiments and in cultures of ZIKV directly from semen. In studies of ZIKV loads in semen, infectious virus could only be cultured from semen with the highest observed viral loads, from 1 × 10^6^ to 2.5 × 10^8^ RNA genome copies of ZIKV per ml (Mead et al., 2018; Medina et al., 2019). We find that SEV are present in semen at an average concentration of 9.85 × 10^12^ per ml. This implies that in semen with the highest viral loads, the ratio of SEV to PFU is around 10^4^–10^5^, where we see no or only partial inhibition of ZIKV (Figures 4B,D,F). As viral load drops but the number of SEV remains constant, this ratio would increase to 10^6^ SEV per virion and greater, where we observed robust inhibition of ZIKV infection in epithelial cells as well as *ex-vivo* vaginal tissues (Figures 4B,D,F), potentially explaining why ZIKV cannot be cultured from semen with lower viral loads. We do not currently know whether semen from other species contains inhibitory SEV. Mouse studies showing sexual ZIKV transmission use IFN-knockout mice which leads to very high viral titers in semen (Uraki et al., 2017), and a study on vaginal inoculation of ZIKV with semen in primates required more than one exposure (Gurung et al., 2020), implying that sexual transmission of ZIKV is still somewhat limited in animal models. One caveat to our studies is that we used semen and SEV from healthy donors rather than ZIKV infected men. We do not know whether the number or quality of SEV in semen from ZIKV infected men differs from that of uninfected individuals, which might influence how they affect ZIKV infection in the recipient mucosa. ZIKV can replicate in cells of the male genital tract and infection can cause prostatitis, hematospermia, and leukocyte shedding in semen (Stassen et al., 2018), suggesting inflammation. Both inflammation and endoplasmic reticulum stress, which active ZIKV replication induces, have been shown to change the number and properties of EV in other systems (Yamamoto et al., 2015; Collett et al., 2018; Xu et al., 2018; Yang et al., 2018). Thus, ZIKV infection may increase the number and diversity of SEV in semen. In addition, increased levels of proinflammatory cytokines in semen from infected men (Mansuy et al., 2019) could trigger an immune response in the recipient mucosa, further preventing infection. Therefore, we postulate that semen from ZIKV infected men could be even more inhibitory than we have observed, but this remains a question for future research.

The finding that SEV potently inhibit ZIKV infection raises the possibility that they play a role in preventing the sexual transmission of other viruses. Other flaviriruses including Chikungunya, West Nile, and Dengue viruses are occasionally detected in semen (Bandeira et al., 2016; Lalle et al., 2018; Gorchakov et al., 2019). Sexual transmission has not been documented for Chikungunya or West Nile. One recent report demonstrates sexual transmission of Dengue (Liew, 2019), though this is likely not a common occurrence. In fact, nucleic acids for 27 different viruses across a broad range of viral families have been found in semen (Salam and Horby, 2017), including most recently SARS-Cov-2 (Li et al., 2020), yet many do not seem to be commonly sexually transmitted. Similarly to what we report here, and to what has been published regarding SEV inhibition of HIV (Madison et al., 2014, 2015), another recent study reports that unfractionated seminal plasma also inhibits sexual transmission of human cytomegalovirus, which is commonly detected in semen (Lippold et al., 2019). This could be because SEV, which are co-delivered at high concentrations with viruses in semen, exert a potent and broad anti-viral effect, a hypothesis which we plan to investigate further. Defining the mechanism of SEV’s antiviral effect could lead to a new ideas and strategies to prevent sexually transmitted viral infections.

## Data Availability Statement

The raw data supporting the conclusions of this article will be made available by the authors, without undue reservation.

## Ethics Statement

The studies involving human participants were reviewed and approved by University of Washington IRB Fred Hutchinson Cancer Research Center IRB. The Ethics Committee waived the requirement of written informed consent for participation.

## Author Contributions

LV, RW, GG, and FH: conceptualization. RW and LV: methodology. RW, GG, YK, UP, and LV: investigation. RW and LV: writing – original draft. UP, YK, GG, and FH: writing – review and editing. LV and FH: funding acquisition. LV, GG, and FH: supervision. All authors contributed to the article and approved the submitted version.

### Conflict of Interest

The authors declare that the research was conducted in the absence of any commercial or financial relationships that could be construed as a potential conflict of interest.
