# Corrigendum: Potent Restriction of Sexual Zika Virus Infection by the Lipid Fraction of Extracellular Vesicles in Semen

**DOI:** 10.3389/fmicb.2021.707875

**Published:** 2021-06-15

**Authors:** Ruofan Wang, Germán G. Gornalusse, Yeseul Kim, Urvashi Pandey, Florian Hladik, Lucia Vojtech

**Affiliations:** ^1^Department of Obstetrics and Gynecology, University of Washington, Seattle, WA, United States; ^2^Division of Allergy and Infectious Diseases, Department of Medicine, University of Washington, Seattle, WA, United States; ^3^Vaccine and Infectious Disease Division, Fred Hutchinson Cancer Research Center, Seattle, WA, United States

**Keywords:** Zika virus, flavivirus, semen, extracellular vesicles, exosomes, female, genital, vagina

In the original article, there was a mistake in the legend for **Figure 4** as published. **Figure 4E** depicts the effect of pre-incubating liposomes with either ZIKV or cells, but the legend mistakenly says SEV rather than liposomes. The correct legend appears below.

**Figure 4**. Lipid content of SEV blocks ZIKV infection. **(A)** The protein content of SEV was denatured by heating at 95° for 8 min. Nanoparticle tracking analysis (NTA) profile comparing heat-treated and untreated SEV showing that the size profiles do not change. Size profiles of liposomes (see part **C**) are also plotted. **(B)** Equal amounts of heat-treated or untreated SEV were incubated with ZIKV virions at a 10^6^ SEV:PFU, then added to epithelial cell lines for 1.5 h before washing and assessing ZIKV binding by ddPCR. As a control, ZIKV virions were also heated at 95° for 8 min. Each line is a separate cell line, points are mean of two replicate wells and error bars are standard deviation. Significance by one-way ANOVA with Tukey's multiple comparisons test. **p > 0.01. **(C)** ZIKV was pre-incubated with 10^6^ SEV or 10^6^ liposomes for 1 h prior to infecting genital epithelial cells. Seventy-two hours post-infection cells were lysed and ZIKV genomes quantified by ddPCR. Each line is the average of duplicate wells from a separate experiment. Reduction in ZIKV genomes was significant by one-way ANOVA with Dunnett's multiple comparisons test. *p < 0.05, and **p < 0.005. **(D)** The data from **C**, and additional conditions with 10^5^ SEV or liposomes, are plotted as percent reduction from ZIKV alone. Each symbol represents the average of two technical wells from a different cell line. The horizontal lines are the mean for each condition. **(E)** Comparison of pre-incubation of either ZIKV or epithelial cells with liposomes on viral infection. Liposomes were pre-incubated with ZIKV (ratio:10^6^ liposome per PFU) or with cells (same amount of liposomes) for 1 h at 37°, then virus was added to cells for 1.5 h. Cells were washed and cell-associated ZIKV genomes quantified by ddPCR. Each symbol represents the average of two technical replicates from different epithelial cell lines. Significance by one-way ANOVA with Dunnett's multiple comparisons test. *p < 0.05.

The authors apologize for this error and state that this does not change the scientific conclusions of the article in any way. The original article has been updated.

