# Supplementary material for: Potent Restriction of Sexual Zika Virus Infection by the Lipid Fraction of Extracellular Vesicles in Semen
Source: Front Microbiol. 2020 Sep 29;11:574054. doi: 10.3389/fmicb.2020.574054 (PMC7550675; doi:10.3389/fmicb.2020.574054)
Supplement: Supplementary file 1 [file Data_Sheet_1.pdf]

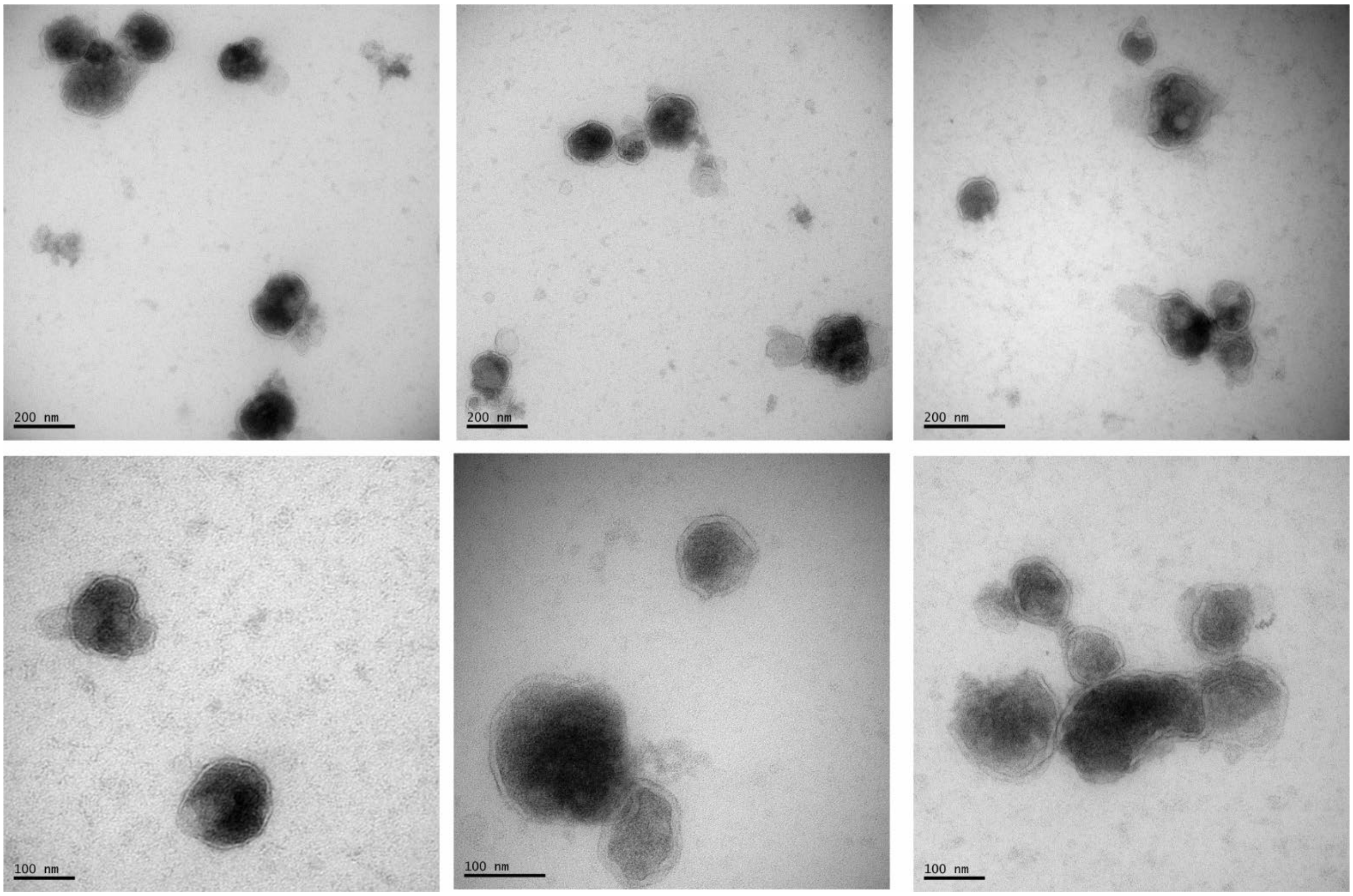

Supplemental figure 1: Transmission electron microscopy images of isolated SEV. Preparation for transmission electron microscopy (EM) analysis was done using the method described by Thery *et al.* (34), except that an airfuge (Beckman-Coulter) was used to deposit fixed samples onto grids. In brief, SE samples were mixed with an equal volume of 4% PFA and deposited by airfuge onto Formvar/Carbon coated EM grids (Ted Pella). Samples were contrasted and embedded by treatment with uranyl-oxalate solution (Electron Microscopy Services), pH 7, for 5 min, followed by methyl-cellulose-uranyl-acetate (Sigma) on ice for 10 min
